# Hemorrhagic gastric ulcer in a patient with Behcet's disease successfully treated with infliximab

**DOI:** 10.1002/deo2.196

**Published:** 2022-12-15

**Authors:** Shun Fujiwara, Ami Kawamoto, Maiko Motobayashi, Shuji Hibiya, Kento Takenaka, Hiromichi Shimizu, Eiko Saito, Toshimitsu Fujii, Masakazu Nagahori, Daisuke Kawata, Natsuka Umezawa, Tadashi Hosoya, Shinsuke Yasuda, Kazuo Ohtsuka, Ryuichi Okamoto

**Affiliations:** ^1^ Department of Gastroenterology and Hepatology Tokyo Medical and Dental University Tokyo Japan; ^2^ Endoscopic Unit Tokyo Medical and Dental University Hospital Tokyo Japan; ^3^ Department of Rheumatology Tokyo Medical and Dental University Tokyo Japan

**Keywords:** Behcet's disease, duodenal ulcers, gastric ulcers, hemorrhage, infliximab

## Abstract

Behcet's disease (BD) is a multisystem immune‐mediated inflammatory disorder that occasionally involves the gastrointestinal tract. Reports on gastrointestinal involvement of BD are relatively rare, of which gastroduodenal involvement is particularly rare. Endoscopic features of gastroduodenal lesions are unknown, and treatment strategies have not been established. In this report, we present the case of a 72‐year‐old female with gastrointestinal BD who presented with extensive gastroduodenal ulcers and hematemesis that were resistant to colchicine and corticosteroid treatment, which were subsequently successfully treated with infliximab. We also review the current literature on the gastroduodenal involvement of BD. Although rare, the case highlights the importance of being aware of upper gastrointestinal manifestations of BD, as well as demonstrating the potential of infliximab to treat corticosteroid‐resistant cases.

## INTRODUCTION

Behcet's disease (BD) was first described by Hulusi Behcet in 1937. It is an immune‐mediated inflammatory disorder characterized by recurrent oral aphthous ulcers, uveitis, skin lesions, and genital ulcers. Intestinal BD can be diagnosed when gastrointestinal symptoms are present, and ulcers are documented objectively. A typical intestinal lesion is characterized by discrete ulcers, usually located in the ileocecal area. Intestinal involvement has been more frequently reported in East Asian countries including Japan and Korea than in other regions, with reports of up to 13.4% of BD patients.^1^ Additionally, intestinal BD with gastroduodenal involvement is extremely rare, with its endoscopic features unknown, and the treatment strategy is yet to be established.^2^ We describe the case of a BD patient who presented with extensive gastroduodenal ulcers and hematemesis, together with a review of the literature.

## CASE REPORT

**FIGURE 1:**
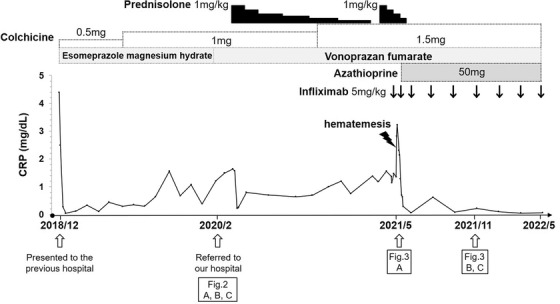
Clinical course of the patient showing relevant treatment and C‐reactive protein (CRP) levels

**FIGURE 2:**
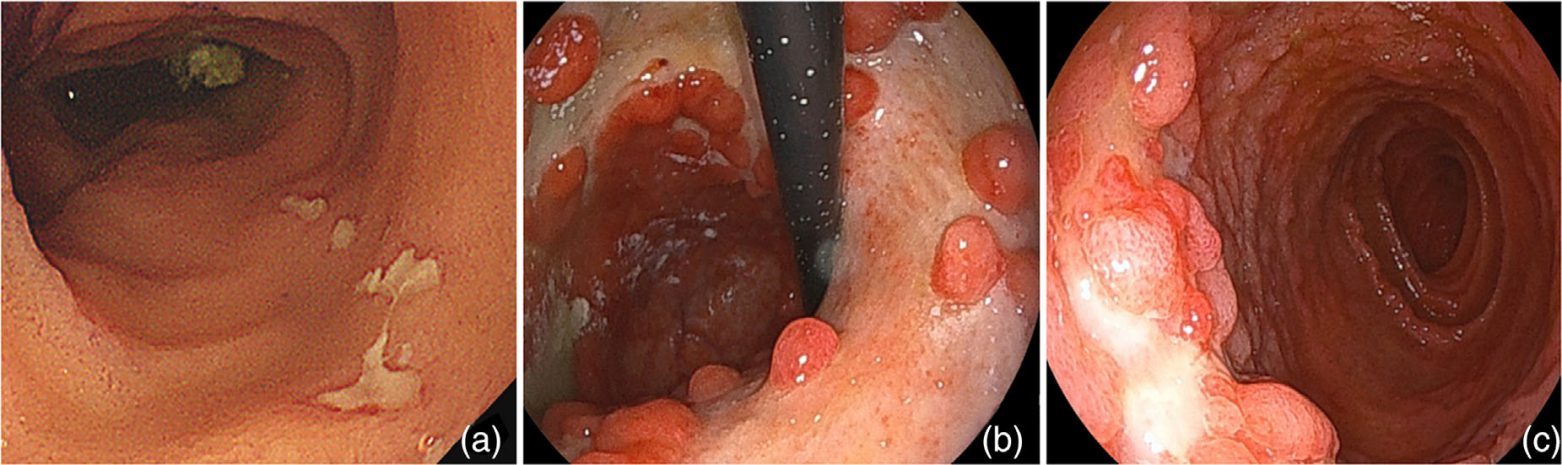
Endoscopic images prior to infliximab treatment. (a) Small discrete ulcers in the terminal ileum. (b) Extensive ulcers in the gastric body and fundus. (c) Irregularly shaped ulcers in the duodenal bulb

**FIGURE 3:**
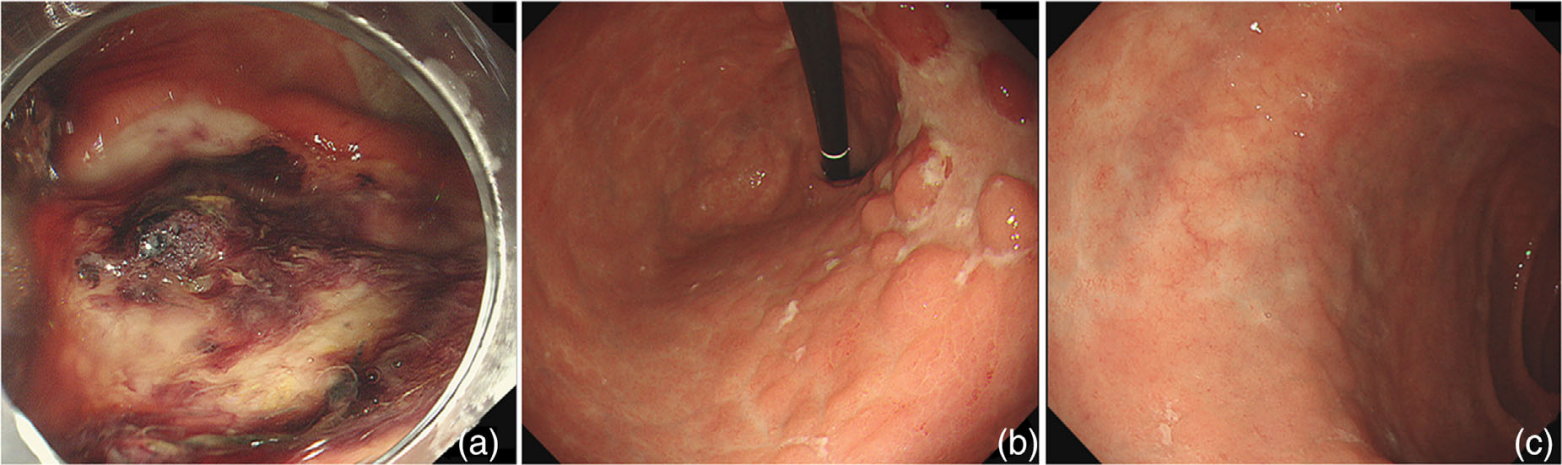
(a) Endoscopic image showing ulcer of the gastric body at the time of hematemesis showing adherent clot. (b,c) Endoscopic images after 6 months of treatment with infliximab. (b) Marked reduction in the size of ulcers of the gastric body and fundus. (c) Ulcer scar of the duodenal bulb

A 72‐year‐old woman with no past medical history presented to her previous physician with fever, anorexia, recurrent oral ulcers, erythema nodosum, and arthritis (Figure 1). Esophagogastroduodenoscopy (EGD) revealed multiple ulcers in the stomach. BD was suspected and colchicine was administered. Although the extra‐gastrointestinal manifestations resolved, the gastric ulcers were resistant to treatment including proton‐pump inhibitors. She was referred to our hospital for further investigation. On presentation, she was apyrexic, with no dermatological, rheumatological, gynecological, or neurological abnormalities on examination. Laboratory examination showed anemia (hemoglobin 11.2 g/dl), elevated C‐reactive protein (1.49 mg/dl), and hypoalbuminemia (albumin 2.5 g/dl; Table S1). We performed a balloon‐assisted enteroscopy revealing small discrete ulcers in the terminal ileum (Figure 2A). EGD showed extensive ulcers throughout the stomach and irregularly shaped ulcers in the duodenal bulb (Figure 2B,C). Serological and repeated pathological analyses revealed no evidence of infections such as *Helicobacter pylori*, tuberculosis, or cytomegalovirus, as well as Crohn's disease or malignancy. Serum gastrin level was normal (100 pg/ml). Human leukocyte antigen B51 was negative. The ulcers were unresponsive to discontinuation of celecoxib, a COX‐2 inhibitor, which had been commenced by her previous physician for arthritis after initial presentation, as well as switching of proton‐pump inhibitors to vonoprazan fumarate, a potassium‐competitive acid blocker. Based on the extra‐gastrointestinal symptoms and endoscopic findings, intestinal BD with gastroduodenal involvement was diagnosed. She was started on treatment with intravenous prednisolone (1 mg/kg, totaling 3152 mg) resulting in temporary improvement of her appetite and C‐reactive protein levels; however, her symptoms relapsed when prednisolone was tapered off and was unresponsive to prednisolone re‐administration (1 mg/kg, totaling 4157 mg). During the course of treatment, she suffered hematemesis and went into hemorrhagic shock. Emergency EGD revealed hemorrhage from one of the gastric ulcers, which was treated with hemostatic forceps (Figure 3A). The gastric ulcers were considered corticosteroid resistant, and infliximab (IFX; 5 mg/kg) was commenced, with subsequent azathioprine addition for combination therapy. IFX was given at 0, 2, and 6 weeks as induction therapy, followed by 8‐weekly maintenance therapy. Her appetite improved markedly, accompanied by a decrease in C‐reactive protein levels, even after corticosteroids were discontinued. After 6 months of treatment with IFX, EGD revealed a marked reduction in the size of gastric ulcers and complete resolution of the duodenal ulcers (Figures 3B,C). At one year, she remains well and continues to undergo 8‐weekly doses of IFX.

## DISCUSSION

BD is a chronic multisystem inflammatory disorder and affects different body systems including oral and genital mucosa, skin, eyes, gastrointestinal tract, joints, vascular, and nervous systems. The ileocecal region is the most frequently reported site of gastrointestinal involvement, followed by the colon. The presence of gastrointestinal lesions presents a risk for intestinal bleeding and perforation. It is therefore important to be aware of their presence and actively control disease activity.

Although it is not common for BD to affect the upper gastrointestinal tract, it has been occasionally reported in recent years, mainly as esophageal lesions. Ye et al. reported that upper gastrointestinal tract involvement may occur either in isolation in 6.82% (84/1232) of cases or together with other digestive tract lesions in 1.62% (20/1232) of cases.^2^ While a report from a single hospital in Taiwan reported the prevalence of gastroduodenal involvement to be as high as 32%, citing possible association with particular human leukocyte antigen genotypes,^3^ a study from Japan only reported duodenal involvement in 2% of BD patients.^4^ Further epidemiological studies should be conducted in order to fully evaluate the prevalence of gastroduodenal involvement in BD. Routine upper gastrointestinal endoscopies may be warranted for BD patients, especially in Asian countries where there seems to be a higher prevalence.

The endoscopic features of BD gastric ulcers are not widely recognized. We searched PubMed (published between 1981 and 2021) and Japan medical abstracts society (published between 1977 and 2021) using the terms “Behcet disease” and “gastric ulcer”. A total of 15 cases were accumulated, and endoscopic features could only be confirmed in nine of the cases (Table 1). There was one case of gastric perforation, and concurrent duodenal involvement was seen in three cases. Reports in the past have described erosions, punched‐out ulcers, and bamboo joint‐like appearances, but there have been no reports of extensive ulcers throughout the entire stomach with ensuing hemorrhage as in our case. More cases need to be accumulated to determine the typical characteristics of gastric BD lesions.

**TABLE 1:** Clinical and endoscopic features of previous reports of gastric Behcet's disease

**Year**	**Author**	**Age/sex**	**Site**	**Endoscopic findings**	**Other gastrointestinal lesions**	**Treatment**
1999	Kimura^1^	32/F	Gastric body and antrum	Erosions	Duodenum and terminal ileum	PPI
2003	Kameda^2^	51/M	Angle and antrum	Punched‐out ulcers	Duodenum and terminal ileum	PPI, 5‐ASA, and PSL
2004	Nojima^3^	69/M	Antrum	Punched‐out ulcers	Terminal ileum	PPI and PSL
2005	Kunisaki^4^	55/M	Angle	Punched‐out ulcers	Esophagus, terminal ileum, and ascending colon	PPI, 5‐ASA, and PSL
2009	Shin^5^	55/M	Gastric body	Punched‐out ulcers	Esophagus, terminal ileum, and colon	PPI, 5‐ASA, Colchicine, PSL, AZA, and surgery
2012	Shimizu^6^	63/F	Gastric body and antrum	Punched‐out ulcers	Splenic flexure	PPI, 5‐ASA, PSL, IFX, and surgery
2015	Hatemi^7^	45/F	Antrum	Gastroduodenal fistulas	Duodenum and anus	PPI and colchicine
2017	Sonoda^8^	68/M	Antrum	Punched‐out ulcers	Terminal ileum	PPI, colchicine, PSL, AZA, and ADA
2018	Tominaga^9^	40/M	Gastric body	Bamboo joint‐like appearances	Esophagus, colon, and rectum	5‐ASA and PSL
	Present case	72/F	Fundus, gastric body, angle, antrum	Extensive ulcers	Duodenum and terminal ileum	PPI/PCAB, colchicine, PSL, AZA, and IFX

Abbreviations: 5‐ASA, 5‐aminosalicylic acid; ADA, adalimumab; AZA, azathioprine; IFX, infliximab; PCAB, potassium‐competitive acid blocker; PPI, proton‐pump inhibitor; PSL, prednisolone.

^1^ Kimura S, Suzuki K, Aizawa T *et al.* A case report of Behcet's disease (incomplete type) complicated with intractable duodenal ulcer causing frequent hematemesis. *Gastroenterol Endosc* 1999; **41**: 2069–75.

^2^ Kameda N, Nakamura S, Hirata N *et al.* A case report of intestinal tract type Behcet's disease with multiple gastroduodenal ulcers and internal fistulas. *Gastroenterol Endosc* 2003; **45**: 144–50.

^3^ Nojima M, Abe T, Igarashi S *et al.* A case of gastric mucosal bridge with Behcet's disease. *Nihon Rinsho Meneki Gakkai Kaishi* 2004; **27**: 177–80.

^4^ Kunisaki R, Kawai T, Shiozawa M *et al.* Beneficial effect of oral mesalazine powder administration on gastric involvement of Behcet's disease. *Dig Endosc* 2005; **17**: 259–62.

^5^ Shin DY, Cheon JH, Park JJ *et al.* Serial episodes of gastric and cecal perforation in a patient with Behcet's disease involving the whole gastrointestinal tract: a case report. *Korean J Gastroenterol* 2009; **53**: 106–10.

^6^ Shimizu Y, Takeda T, Matsumoto R *et al.* Clinical efficacy of adalimumab for a postoperative marginal ulcer in gastrointestinal Behcet disease. *Nihon Shokakibyo Gakkai Zasshi* 2012; **109**: 774–80.

^7^ Hatemi I, Hatemi G, Erzin YZ *et al.* Double pylorus in a patient with Behcet's syndrome. *Clin Exp Rheumatol* 2015; **33**: 138–40.

^8^ Sonoda A, Ogawa R, Mizukami K *et al.* Marked improvement in gastric involvement in Behcet's disease with adalimumab treatment. *Turk J Gastroenterol* 2017; **28**: 405–7.

^9^ Tominaga K, Kamimura K, Takahashi K *et al.* A case of Behcet's disease with various gastrointestinal findings. *Clin J Gastroenterol* 2018; **11**: 354–8.

In clinical settings, 5‐aminosalicylic acid, systemic corticosteroids, and immunosuppressive agents such as thiopurines are known to be effective treatments for intestinal BD. The anti‐tumor necrosis factor alpha agents IFX and adalimumab (ADA) have recently been approved for the treatment of patients with intestinal BD affected by severe disease or resistant to existing treatments in Japan. IFX therapy showed a complete response rate of 61% at 54 weeks post‐induction, while that of ADA was 60% at 52 weeks post‐induction.^5^, ^6^ While it has been shown to be effective in ileal involvement, its effectiveness in gastric involvement has not been reported. In the previous article search, prednisolone was used in 78% of the nine cases of BD with gastric lesions. Anti‐tumor necrosis factor alpha antibodies were used in two cases. ADA was used in one corticosteroid‐dependent case, with successful corticosteroid withdrawal and mucosal healing. IFX was used in another corticosteroid‐resistant case, but improvement could not be seen and the patient required a total gastrectomy. Possibly owing to the intravenous route of administration, IFX is known to produce a more rapid effect compared to ADA, especially in ulcerative colitis patients. Although the speed of induction for BD patients has not been as widely studied compared to inflammatory bowel disease patients, a prospective single‐arm phase 3 study including intestinal BD patients showed that IFX showed clinical response as early as 2 weeks after administration.^5^ Because our case developed hemorrhagic shock requiring rapid response, IFX was chosen over ADA. Combination therapy involving immunomodulators and IFX is widely used to suppress neutralizing antibody production and improve therapeutic efficacy. Although data are limited for BD patients, one study involving intestinal BD patients showed that concomitant immunomodulator use was independently associated with a sustained response to IFX.^7^ It has also been reported that the use of combination therapy does not necessarily increase the risk of complications in elderly patients compared to younger patients with Crohn's disease.^8^ Because of the severe nature of our patient's presentation, we weighed the risks of complications of combination therapy against the benefit of relapse prevention, and after carefully consulting the patient regarding these points, started the patient on azathioprine. The patient is free of any complications including infections as of date. Concomitant immunomodulator use with IFX was one of the differences between our case and the previously reported case of gastric BD which was unresponsive to IFX treatment and subsequently required gastrectomy. Further studies are needed to evaluate the efficacy of combination therapy in gastroduodenal BD cases.

We described the first‐ever case of BD with extensive gastroduodenal ulcers with ensuing hemorrhage, in which combination therapy with IFX and azathioprine was effective. Although our report is limited to a single case, it adds insight into the spectrum of gastrointestinal manifestations of BD, as well as showing potential for IFX in severe cases.

## CONFLICT OF INTEREST

None.

## Supporting information




**Table S1** Laboratory data at the time of referral to our hospital
